# The impact of COVID-19 on work-related mental health claims of healthcare workers in British Columbia: an interrupted time series analysis

**DOI:** 10.1192/j.eurpsy.2023.994

**Published:** 2023-07-19

**Authors:** M. A. Banal, J. Puyat

**Affiliations:** School of Population and Public Health, University of British Columbia, Vancouver, Canada

## Abstract

**Introduction:**

Healthcare workers (HCW) have been at the forefront of providing care since the COVID-19 pandemic. In addition to physical demands, HCW are also vulnerable to mental health conditions due to the nature of their work. As a result, absenteeism among HCW is inevitable. In Canada, mental disorders caused by a stressor at work results in a work-related claim provided it meets the criteria of a governing worker’s compensation agency. While the literature points to varying prevalence rates of mental health illnesses among HCW, it remains unknown how the COVID-19 pandemic affected the number of work-related mental health claims in this population.

**Objectives:**

To help fill this gap in knowledge, we will conduct this study that aims to determine the impact of the COVID-19 pandemic on the number of work-related mental health claims among HCW.

**Methods:**

We will utilize deidentified individual data from a worker’s compensation agency in all of British Columbia. Mental health claims will be identified using an indicator for mental health. Diagnoses for mental health conditions in these claims are ascertained by a psychologist or psychiatrist. Differences in the number of mental health claims between HCW and non-HCW before (January - February 2020) and after (March 2020 - December 2021) the pandemic will be estimated using interrupted time series analysis.

**Results:**

The findings will inform disability case managers, healthcare providers, and employers the importance of identifying appropriate work accommodations, return to work programs and additional mental health supports for HCW under mental health claims. Healthcare unions in British Columbia can use the findings to advocate for better work accommodations and mental health support for HCW.

**Conclusions:**

Further understanding the complications of long-term effects of COVID-19 on mental health of HCW will inform workforce planning and patient care.

**Disclosure of Interest:**

None Declared

